# Tibial plateau fractures (AO type B3) combined with tibial tubercle fracture: Case report and review of the literature

**DOI:** 10.1097/MD.0000000000012015

**Published:** 2018-09-07

**Authors:** Lei Tan, Yan-Hui Li, Yuying Li, Tong Lin, Dong Zhu, Da-Hui Sun

**Affiliations:** a Department of Orthopedic Trauma; b Department of Cardiology and Echocardiography; c Department of Hematology, The First Hospital of Jilin University, Changchun, China.

**Keywords:** tibial plateau fractures, tibial tubercle fractures, Type B3

## Abstract

**Rationale:**

Tibial tuberosity fractures most often occur in the adolescents. Fracture of tibial tuberosity in adults is extremely rare with only 5 reported cases till date. Tibial plateau fractures combined with tibial tubercle fractures are not common.

**Patient concerns:**

We report here a type B3 tibial plateau fracture (AO classification) with a concomitant fracture of tibial tuberosity.

**Diagnoses:**

Anteroposterior and lateral knee view radiographs revealed a complex comminuted fracture of the right tibial plateau (AO Type B3; Schatzker Type IV) with tibial tubercle fracture. Three-dimensional computed tomography (CT) showed that the tibial media plateau was split into 2 pieces in the sagittal plane, along with the isolated tibial tubercle.

**Interventions:**

The open procedure was performed first and a standard posteromedial approach for medial and posteromedial tibial plateau fracture was used with double locking plate fixation. The tibial tuberosity was fixed with a cortical screw.

**Outcomes:**

The patient showed full range of motion in right knee after 8 weeks. The patient was allowed full weight bearing at 4 months. Eight months after operation, he was asymptomatic, showed a full range of motion and good strength. He had returned to work with no limitations.

**Lessons:**

Fractures of the partial tibial plateau combined with tibial tubercle are present and should not be ignored. Accurate diagnosis and proper treatment will help achieve favorable outcomes in these patients.

## 1 Introduction

Tibial tuberosity fractures most often occur in the adolescents.^[1]^ Fracture of tibial tuberosity in adults is extremely rare with only 5 reported cases till date.^[2–6]^ Tibial plateau fractures combined with tibial tubercle fractures are not common. In the classification system of Schatzker, there is no specific description of the fracture of the tibial tuberosity. In the arbeitsgemeinschaftfür osteosynthesefragen (AO) type, both type A1 and type C include fracture of the tibial tuberosity, emphasizing the need for attention and treatment. The AO type B fracture, which is a partial articular fracture, does not include a subtype with concomitant tibial tuberosity fracture. Does this mean that this kind of fracture does not occur with a concomitant fracture of tibial tubercle? We report here a type B3 tibial plateau fracture (AO classification) with a concomitant fracture of tibial tuberosity. To the best of our knowledge, this is the first report of this type of injury.

## 2 Case report

The institutional review board at the First Hospital of Jilin University approved this work and informed consent of the patient was obtained. A 40-year-old man presented to our emergency department with a closed injury to the right knee sustained in a motorcycle accident 3 hours ago. On physical examination, he had a swollen, tender right knee joint with restricted motion; there were no signs of compartment syndrome. Sensation and distal limb pulses were intact. The limb was initially splinted and subjected to radiological evaluation.

**Figure 1:**
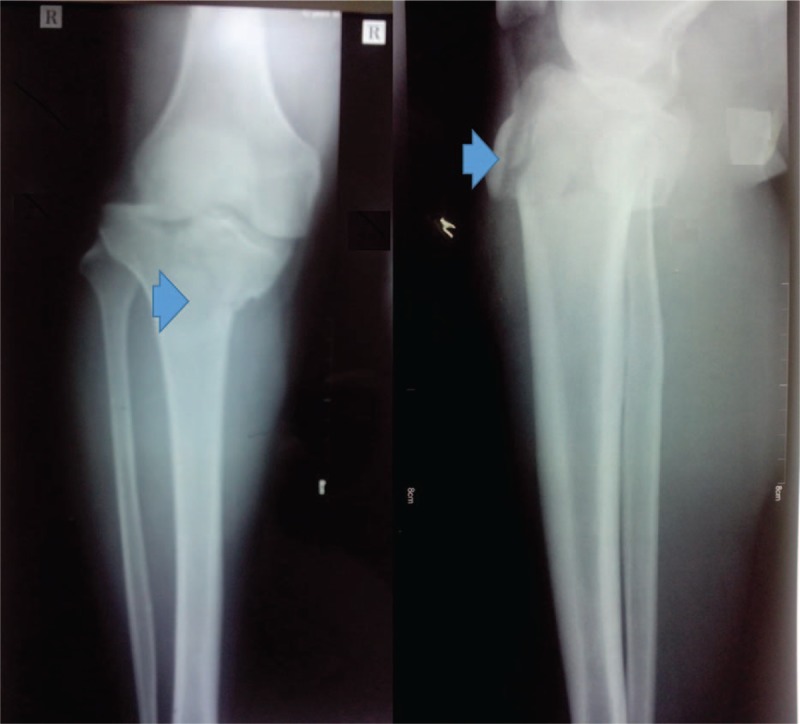
Preoperative anteroposterior (AP) and lateral view of the knee: a complex comminuted fracture of the right tibial plateau (AO Type B3; Schatzker Type IV) with tibial tubercle fracture (blue arrow head). AO = arbeitsgemeinschaftfür osteosynthesefragen, AP = anteroposterior.

**Figure 2:**
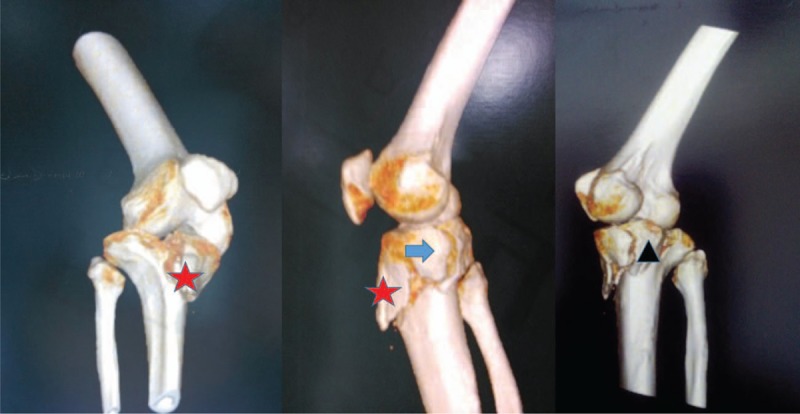
Three-dimensional computed tomography (CT) showing the tibial media plateau split into 2 pieces in the sagittal plane (black triangle and blue arrow), and the depression of the lateral plateau, the isolated tibial tubercle (red star). CT = computed tomography.

Anteroposterior and lateral knee view radiographs revealed a complex comminuted fracture of the right tibial plateau (AO Type B3; Schatzker Type IV) with tibial tubercle fracture (Fig. 1). Three-dimensional computed tomography (CT) showed that the tibial media plateau was split into 2 pieces in the sagittal plane, along with depression of the lateral plateau, the isolated tibial tubercle; most of the lateral plateau was still connected with the metaphysis (Fig. 2).

**Figure 3:**
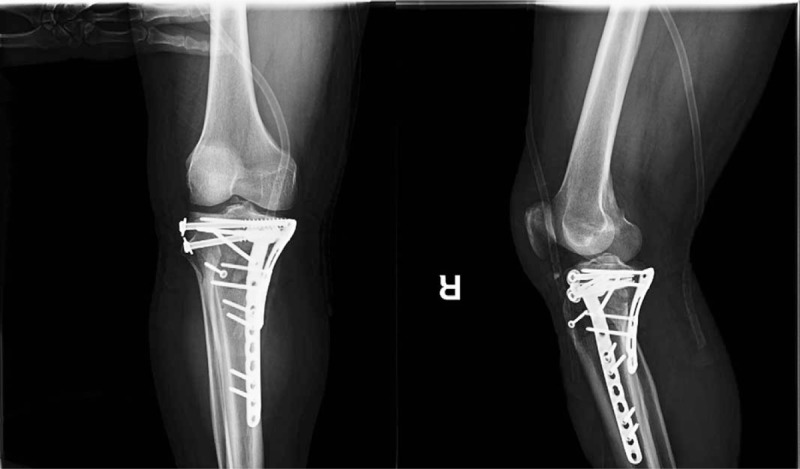
Postoperative anteroposterior (AP) and lateral view of the knee. AP = anteroposterior.

The open procedure was performed first and a standard posteromedial approach for medial and posteromedial tibial plateau fracture was used with double locking plate fixation. After reduction of the articular surface through a standard anterolateral approach, the lateral tibial plateau depression hole was filled with allogeneic bone graft, and then fixed with two 6.5 mm cannulated screws. Then the tibial tuberosity was fixed with a cortical screw (Fig. 3). Elevation of the articular surface was confirmed on immediate postoperative x-rays.

One week after the surgery, knee bending and quadriceps exercises were initiated. The patient showed full range of motion in right knee after 8 weeks. The patient was allowed full weight bearing at 4 months. Eight months after operation, he was asymptomatic, showed a full range of motion and good strength. He had returned to work with no limitations.

## 3 Discussion

Tibial tubercle fractures, which represent a disruption of the quadriceps mechanism, are not rare, particularly in the setting of Schatzker type V and VI fractures. Maroto et al^[7]^ retrospectively reviewed a prospectively compiled orthopedic trauma database and identified 392 bicondylar fractures of the tibial plateau, in which 85 tibial tubercle fractures (21.6%) were identified in 84 patients. But for Schatzker type I to IV fractures combined with tibial tubercle fractures, no one has yet reported. In the present case, a Schatzker IV or AO type B3 tibial plateau fracture was observed along with a fracture of the tibial tuberosity. To the best of our knowledge, this is the first report of this type of injury.

Tibial tubercle fractures may easily be missed in complex injuries. The examiner is distracted by the more attractive displaced fragments of the plateau, whose comminution may obscure the tubercle fragment. A poorly performed knee lateral radiograph is liable to miss the fracture plane entirely. If a tibial tubercle fracture is missed and left unfixed, the patient will rehabilitate poorly due to pain; moreover, displacement of the fragment may result in an extension lag that is unrecoverable. In addition, failure to address the tibial tubercle component of a tibial plateau fracture has been shown to result in nonunion of the tibial tubercle fracture.^[8]^

Therefore, we should be alert to the fact that AO type B plateau fracture may be accompanied by a fracture of the tibial tubercle. In particular, attention should be paid to the integrity of the tibial tubercle and the level of the patella while reading the x-ray film. Three-dimensional CT is indispensible in such cases. Sagittal plane two- and three-dimensional reconstruction images may best demonstrate tibial tubercle fractures.

The tibial tubercle is the end point of the patellar ligament. In order to maintain the integrity of the extensor mechanism, the tuberosity fracture will need anatomical reduction to ensure restoration of knee function.^[9,10]^ Methods for treatment of tibial tuberosity fracture include use of tension band wire, independent cortical lag screws or screws placed through a plate, which serve to increase the surface area covered by the screws.^[7,9,11–15]^ The choice of treatment should be guided by the morphology of tibial tuberosity fracture and the characteristics of the tibial plateau fracture. When the tibial tubercle fracture is a single large noncomminuted fragment, use of tension band wire or fixation of the tubercle to the posterior tibial cortex with one or more lag screw is the traditionally preferred surgical option.^[2,6,13,15]^ In cases where comminution of the tibial tubercle fracture or posterior tibial cortex is present, the cortex of the posterior tibia does not always offer good purchase for screw fixation; plate and screws are preferred in such cases.^[7]^ The plate in effect serves to resist the pull of the extensor mechanism on the tibial tubercle. This allows rigid fixation of the tubercle fragment. In this study, the treating surgeon selected screw to fix the fracture.

Maroto et al^[7]^ suggested the tibial tubercle should also be conformed and treated when a suspected or incomplete fracture exists, so as to avoid displacement or malunion in the postoperative period with early mobilization of the knee. As the tibial tubercle has relatively sparse overlying soft tissue, care must be taken to protect the soft tissue envelope during exposure and fixation. Further, it may be necessary to remove the fixation device after bony union has occurred.

## 4 Conclusion

Fractures of the partial tibial plateau combined with tibial tubercle are present and should not be ignored. Accurate diagnosis and proper treatment will help achieve favorable outcomes in these patients.

## Author contributions

**Conceptualization:** Da-Hui Sun.

**Data curation:** Tong Lin.

**Investigation:** Yuying Li.

**Resources:** Dong Zhu.

**Writing – original draft:** Lei Tan.

**Writing – review & editing:** Yan-Hui Li, Da-Hui Sun.

## References

[1] Frey S Hosalkar H Cameron DB Tibial tuberosity fractures in adolescents. J Child Orthop 2008;2:469–74. 19308544 10.1007/s11832-008-0131-z PMC2656872

[2] Kanawati AJ Lorentzos P Avulsion fracture of the tibial tubercle in an adult treated with tension-band wiring: a case report. Internet J Orthop Surg 2010;18:1–4.

[3] Mounasamy V Brown TE Avulsion fracture of the tibial tuberosity with articular extension in an adult: a novel method of fixation. Eur J Orthop Surg Traumatol 2008;18:157–9.

[4] Vella D Peretti G Fra F One case of fracture of the tibial tuberosity in the adult. La Chirurgia Degli Organi Di Movimento 1992;77:299–301. 1424965

[5] Liu YP Hao QH Lin F Tibial tuberosity avulsion fracture and open proximal tibial fracture in an adult: a case report and literature review. Medicine 2015;94:1684–0. 10.1097/MD.0000000000001684 PMC4616828 26426669

[6] Aterkar VM Mahajan UD Somani AM Tibial tuberosity avulsion fracture in an adult—a rare case report. Int J Med Res Health Sci 2014;3:1016.

[7] Maroto MD Scolaro JA Henley MB Management and incidence of tibial tubercle fractures in bicondylar fractures of the tibial plateau. Bone Joint J 2013;95-B:1697–702. 24293602 10.1302/0301-620X.95B12.32016

[8] Phisitkul P Mckinley TO Nepola JV Complications of locking plate fixation in complex proximal tibia injuries. J Orthop Trauma 2007;21:83. 17304060 10.1097/BOT.0b013e318030df96

[9] Stanitski CL Acute tibial tubercle avulsion fractures. Oper Tech Sports Med 1998;6:243–6.

[10] Mckoy BE Stanitski CL Acute tibial tubercle avulsion fractures. Orthop Clin North Am 2004;24:181. 10.1016/s0030-5898(02)00061-5 12974489

[11] Bauer T Milet A Odent T Avulsion fracture of the tibial tubercle in adolescents: 22 cases and review of the literature. Revue Chirurgie Orthop Réparatrice Lappareil Moteur 2005;91:758–67. 10.1016/s0035-1040(05)84487-3 16552998

[12] Abalo A Akakpo-Numado KG Dossim A Avulsion fractures of the tibial tubercle. J Orthop Surg 2008;16:308–11. 10.1177/230949900801600308 19126896

[13] Cobey MC Lag-screw fixation in fractures of the tibial tuberosity. J Bone Joint Surg Am 1946;28:273. 21020229

[14] Ergün M Taşkiran E Ozgürbüz C Simultaneous bilateral tibial tubercle avulsion fracture in a basketball player. Knee Surg Sports Traumatol Arthrosc 2003;11:163. 12774153 10.1007/s00167-003-0342-2

[15] Tang Y Zhang YT Fu QG Application of cannulated compression screws for the treatment of tibial tubercle avulsion fractures of Ogden type III in adolescents. China J Orthop Traumatol 2013;26:717–9. 24416899

